# Editorial: Current trends in environmental psychology, volume II

**DOI:** 10.3389/fpsyg.2023.1265274

**Published:** 2023-08-22

**Authors:** Oriana Mosca, Ferdinando Fornara, Stefano Mastandrea, Ylenia Passiatore, Mauro Sarrica, Massimiliano Scopelliti, Giuseppe Carrus

**Affiliations:** ^1^Department of Education, Psychology and Philosophy, University of Cagliari, Cagliari, Italy; ^2^Experimental Psychology Laboratory, Department of Education, Roma Tre University, Rome, Italy; ^3^Department of Communication and Social Research, Sapienza University of Rome, Rome, Italy; ^4^Department of Human Studies, LUMSA University, Rome, Italy

**Keywords:** environmental psychology, transitions to sustainability, COVID-19, place attachment, organizational environments, outdoor environments, ecological behaviors, digital environments

This Research Topic, “*Current trends in environmental psychology - volume II*”, is associated with the 3rd International Conference of Environmental Psychology (ICEP 2021), which was held in Siracusa, Italy, from the 4th to 9th October 2021 and it is the natural prosecution, and completion, of the volume I of the same Research Topic (see also De Gregorio et al., 2023 for an overview). The opening of the Research Topic was made during a very difficult time: the threat of a global pandemic/syndemic from the COVID-19 viral infection was a dominant international concern and was changing drastically living conditions at a global level. Since its emergence in early 2020, the COVID-19 pandemic has monopolized public opinion: the permanent threat to citizens' health and safety, the sweeping measures that national governments have adopted, and their economic repercussions, have cast some shadow on other relevant and urgent problems outlined by environmental scientists, such as global warming and climate change, and might continue to affect how they are seen and interpreted. The spread of the disease gave rise to a condition characterized by the implementation of physical distancing measures, entailing human disconnection, isolation, and increased reliance on virtual interactions, as well as a greater emphasis on remote work through information and communication technologies (ICT). These transformations might have occurred abruptly, perhaps forcefully, perhaps in a shock and awe way, but surely at the expense of diseases, panic, mental health issues, and in the worst-case scenario loss of life. Nowadays, these emergency measures are overcome. In fact, while we are writing this editorial we are breathing an air of hope for the future, profoundly aware of the deep scars left on our world, serving as a permanent reminder and inspiration for the continuous adjustment of the implementation of complex solutions to complex people-places relationship issues. On May 5th 2023, the World Health Organization officially declared the end of the pandemic. However, while addressing this in a post-pandemic world, negative repercussions are emerging, with a significant portion of the global population residing and working in confined spaces. The COVID-19 challenge was finally overcome thanks to an enourmous global effort of people, governments, scientific institutions and organizations (including OA publishers such as Frontiers). Some mistakes were also made, including a lack of coordination, equity, and solidarity at the global level in some cases. That means that existing tools and technologies were not always used at their best to answer the complex societal issues posited by the pandemic.

The aim of this Research Topic was the promotion of the scientific dialogue over the most recent empirical findings and theoretical advances in environmental psychological science, and to build evidence-based knowledge and innovative approaches to understanding the relationship between humans and their socio-physical environments.

The influence of COVID 19 in environmental issues was highlighted, for example, by the study of Bertolotti et al. showing that people's attitudes toward the environmental crisis can be influenced also by counterfactuals arguments related to the expenses incurred during the COVID-19 pandemic, suggesting a potential conflict between economic and health issues. This suggests a connection between public perception of the pandemic and climate change, even though they appeared initially unrelated. The echo deriving from the pandemic period is likely to impact communication efforts related to climate change actually and in the future as well. The authors showed that communication strategies are crucial in motivating individuals to responsibly engage in the environmental domain.

There was a variety of built environments investigated, like homes and offices with a different focus: Nartova-Bochaver et al. analyzed the home environment, considering the construct of home attachment, which is especially important for students away from their hometown as one of the most mobile social group. The authors validated cross-culturally the Short Home Attachment Scale (SHAS) in a student sample from five countries (Armenia, India, Indonesia, Russia, and Ukraine), offering a psychometric contribution in line with recent suggestions by Tam and Milfont (2020). The authors, acknowledging that human–environment interactions are culture-bound, outlined the vital importance for environmental psychology research to incorporate the understanding of culture into theoretical analyses and empirical investigations.

Different authors devoted their attention to organizational settings and environmental issues: Chen and Wu in order to investigate employees' green behavior, explored the interaction effect of green Human Resource Management practices and green transformational leadership on employees' green behavior, acknowledging the intermediary role of green mindfulness and the regulatory effect of green self-efficacy. This contribution could help both the academic community to better understand how green-related contextual factors jointly influence environmental behaviors and in providing successful recommendations to corporate managers. Zhu et al., starting from the consideration that current urban lifestyle lead people to spend more and more time in office settings and that prolonged periods in an uncomfortable environment can have negative impacts on employees' wellbeing, analyzed the aesthetic evaluations of different office types of furniture. The authors specifically examined the incorporation of wood in office furniture as a means to create a healthy environment, suggesting that the use of wood in office spaces has been found to effectively alleviate mental fatigue among employees, creating more pleasant, desirable and restorative offices. Ma et al. proposed a moderated mediation model to explain when and how tourism service firms can promote employee retention by considering Green Talent Management strategies. The findings of this study provide meaningful insights for managers and service firms in the tourism industry.

Outdoor environments were also considered with different approaches: Bruzzese et al. investigated civil society's perception and knowledge toward Forest Ecosystem Services (FES) and how these changed in the post-COVID era. The authors presented a very informative case study: they conducted a choice experiment with individuals intercepted in the Argentera Valley, in the Western Italian Alps, highlighting a strong interest in biodiversity and cultural services, such as landscape aesthetic quality and psychophysical health. These findings could be useful to optimize the matching of supply and demand and to provide more robust information for promoting the participatory and shared decision-making process in forest planning and management. The importance of nature was investigated also with a theoretical contribution: Prins et al. conducted a systematic review and meta-ethnography of qualitative research on the value of play in nature-based compared to non-nature-based environments, and its implications for the developmental outcomes of young children (2–8 year). Their study showed that playing in nature-based environments supports young children's healthy global development, i.e. physical, social-emotional, motor, and cognitive. These results could be a further interesting insight to understand the dynamics and processes of humans-nature connectedness.

Other studies have applied socio-environmental theories and models to a wide range of issues and topics: Haji and Hayati aimed to provide a comprehensive theoretical framework in the field of analyzing conflict behavior among rangeland exploiters in Iran. Specific environmental psychological theories, such as the Norm Activation Theory, the Value Beliefs Norms Theory, or the Theory of Planned Behavior, were found a suitable framework to explain conflict behavior in rangeland exploitation contexts. Likewise, using the Social Identity Model of Collective Action, Valizadeh et al. investigated Iranian farmers' intentions to participate in Aquifer Storage and Recovery, an innovative and alternative method for the sustainable management of water resources. The authors highlighted the need to consider the formation of social identity and the consideration of “we” thinking systems as the best strategy for aquifer storage and recovery. The role of personality factors in shaping pro-environmental behaviors was also investigated by other studies: Haefner et al. investigated the mediating role of animal-related ethical values in the association between Big Five Personality traits, animal-related ethical values, and different types of meat consumption (i.e., beef, poultry, and fish), providing useful information for susrtainable dietary change.

Finally, digital environment studies could not miss the call in this RT: Suseno and Hastjarjo investigated the role of simulated natural environments in virtual reality and 2D video in reducing stress.

Researchers from Europe (in particular, Italy, Germany, and the Netherlands), Russia, China, Iran, and, Indonesia have contributed to the co-construction of a collective scientific endeavor that at this moment has collected about 25,000 views across 11 different papers.

The richness and diversification of the published articles were the natural answers to complex questions like the ones presented in the call for papers of our Research Topic. A commitment to trans-disciplinarity was emerging and it could be seen as a form of cooperative research among the different parts of society, professionals, and academia (Pohl and Hadorn, 2008), enabling the blurring, and then the transcending, of the boundaries between different disciplines. Trans-disciplinarity, in its hybrid and non-linear nature, enables it to transcend and indeed incorporate any academic disciplinary structure. We like to believe that also our RT could offer a small but significant contribution toward this direction.

## Author contributions

OM: Conceptualization, Writing—original draft, Writing—review and editing. FF: Conceptualization, Supervision, Writing—review and editing, Writing—original draft. SM: Conceptualization, Supervision, Writing—review and editing, Writing—original draft. YP: Conceptualization, Writing—review and editing, Writing—original draft. MSa: Conceptualization, Supervision, Writing—review and editing, Writing—original draft. MSc: Conceptualization, Supervision, Writing—review and editing, Writing—original draft. GC: Conceptualization, Funding acquisition, Project administration, Supervision, Writing—review and editing, Writing—original draft.
